# Nanoarchaea: representatives of a novel archaeal phylum or a fast-evolving euryarchaeal lineage related to Thermococcales?

**DOI:** 10.1186/gb-2005-6-5-r42

**Published:** 2005-04-14

**Authors:** Celine Brochier, Simonetta Gribaldo, Yvan Zivanovic, Fabrice Confalonieri, Patrick Forterre

**Affiliations:** 1 EA EGEE (Evolution, Génomique, Environnement) Université Aix-Marseille I, Centre Saint-Charles, 3 Place Victor Hugo, 13331 Marseille, Cedex 3, France; 2 Unite Biologie Moléculaire du Gène chez les Extremophiles, Institut Pasteur, 25 rue du Dr Roux, 75724 Paris Cedex 15, France; 3 Institut de Génétique et Microbiologie, UMR CNRS 8621, Université Paris-Sud, 91405 Orsay, France

## Abstract

**Background:**

Cultivable archaeal species are assigned to two phyla - the Crenarchaeota and the Euryarchaeota - by a number of important genetic differences, and this ancient split is strongly supported by phylogenetic analysis. The recently described hyperthermophile *Nanoarchaeum equitans*, harboring the smallest cellular genome ever sequenced (480 kb), has been suggested as the representative of a new phylum - the Nanoarchaeota - that would have diverged before the Crenarchaeota/Euryarchaeota split. Confirming the phylogenetic position of *N. equitans *is thus crucial for deciphering the history of the archaeal domain.

**Results:**

We tested the placement of *N. equitans *in the archaeal phylogeny using a large dataset of concatenated ribosomal proteins from 25 archaeal genomes. We indicate that the placement of *N. equitans *in archaeal phylogenies on the basis of ribosomal protein concatenation may be strongly biased by the coupled effect of its above-average evolutionary rate and lateral gene transfers. Indeed, we show that different subsets of ribosomal proteins harbor a conflicting phylogenetic signal for the placement of *N. equitans*. A BLASTP-based survey of the phylogenetic pattern of all open reading frames (ORFs) in the genome of *N. equitans *revealed a surprisingly high fraction of close hits with Euryarchaeota, notably Thermococcales. Strikingly, a specific affinity of *N. equitans *and Thermococcales was strongly supported by phylogenies based on a subset of ribosomal proteins, and on a number of unrelated molecular markers.

**Conclusion:**

We suggest that *N. equitans *may more probably be the representative of a fast-evolving euryarchaeal lineage (possibly related to Thermococcales) than the representative of a novel and early diverging archaeal phylum.

## Abstract 2

An analysis of the position of *Nanoarcheum equitans* in the archaeal phylogeny using a large dataset of concatenated ribosomal proteins from 25 archaeal genomes suggests that *N. equitans* is likely to be the representative of a fast-evolving euryarchaeal lineage.

## Background

Despite a ubiquitous distribution [1] and a diversity that may parallel that of the Bacteria (for a recent review see [2]), the Archaea still remain the most unexplored of life's domains. Whereas 21 different phyla are identified in the Bacteria (National Center for Biotechnology Information (NCBI) Taxonomy Database, as of October 2004 [3]), known cultivable archaeal species fall into only two distinct phyla - the Crenarchaeota and the Euryarchaeota [4] - on the basis of small subunit rRNA (SSU rRNA) (NCBI Taxonomy Database, as of October 2004 [3]). A number of non-cultivated species that do not group with either Crenarchaeota or Euryarchaeota have been tentatively assigned to a third phylum, the Korarchaeota [5]. However, this group may be artefactual, as well as that formed by other environmental 16S rRNA sequences [2].

The Crenarchaeota/Euryarchaeota divide indicated by SSU rRNA phylogenies is strongly supported by comparative genomics, as a number of genes present in euryarchaeal genomes are missing altogether in crenarchaeal ones and vice versa. These differences are not trivial, as they involve key proteins involved in DNA replication, chromosome structure and replication. For example, the Crenarchaeota lack both DNA polymerases of the D family and eukaryotic-like histones, which are present in the Euryarchaeota [6,7]. Similarly, replication protein RPA and cell-division protein FtsZ remain exclusive to the Euryarchaeota [8], while only the Crenarchaeota harbor the ribosomal protein S30 (COG4919). This suggests that members of these two archaeal subdomains may employ critically different molecular strategies for key cellular processes. The distinctiveness of the phyla Euryarchaeaota and Crenarchaeota is further strengthened by phylogenetic analysis ([9,10] and this work) and is likely to remain unaffected even when additional cultivable species will be defined. Such a dramatic split is intriguing as it may be more profound than that separating the different bacterial phyla and leaves open different scenarios for the origin of these important differences during early archaeal evolution.

Karl Stetter and his colleagues recently described a novel archaeal species - *Nanoarchaeum equitans *- representing the smallest known living cell [11]. This tiny hyperthermophile grows and divides at the surface of crenarchaeal *Ignicoccus *species and cannot be cultivated independently, indicating an obligate symbiotic, and possibly parasitic, life style [12]. Sequencing of the *N. equitans *genome revealed the smallest cellular genome presently known (480 kb) and raised fascinating questions regarding the origin and evolution of this archaeon [13]. Indeed, in contrast to typical genomes from parasitic/symbiotic microbes [14-16], that of *N. equitans *does not show any evidence of decaying genes and contains a full complement of tightly packed genes encoding informational proteins [13]. This suggests that the establishment of the dependence-relationship between *N. equitans *and *Ignicoccus *is probably very ancient. In a phylogeny of 14 archaeal taxa based on a concatenation of 35 ribosomal proteins and rooted by eukaryotic sequences, *N. equitans *emerged as the first archaeal lineage, that is, before the divergence of the two main archaeal phyla, the Euryarchaeota and the Crenarchaeota [13]. This is consistent with the early emergence of *N. equitans *in a phylogeny based on SSU rRNA [12], and with the proposal that *N. equitans *should be considered as the representative of a novel and very ancient archaeal phylum, the Nanoarchaeota [11].

Testing the phylogenetic position of *N. equitans *is thus crucial to deciphering the history of the archaeal domain. For instance, if the divergence of this lineage indeed preceded the divergence of Euryarchaeota and Crenarchaeota, features common to *N. equitans *and any other archaeal taxa could probably be considered as ancestral characters (provided that lateral gene transfers (LGTs) are excluded). For example, the most parsimonious interpretation for the presence in the genome of *N. equitans *of all those genes that are otherwise found in the Euryarchaeota only [13] is that all these proteins were present in the last archaeal ancestor and were subsequently lost in the Crenarchaeota. However, the hypothesis of an early divergence of the Nanoarchaeota should be treated with caution. There are now several examples in which fast-evolving taxa are mistakenly assigned to early branches because of a long branch attraction (LBA) artifact due to their high evolutionary rates [17], especially when a distant outgroup is used [18-21]. Similarly, since adaptation to a symbiotic or parasitic life style may have accelerated its evolutionary rate, the basal position of *N. equitans *in phylogenetic analyses using distant eukaryotic sequences as the outgroup [13] may be strongly affected by LBA.

We tested the position of *N. equitans *in the archaeal phylogeny by using a dataset of concatenated ribosomal proteins larger than that used by Waters and colleagues [13], a much broader taxonomic sampling, and without including any outgroup in order to reduce LBA. By applying phylogenetic approaches that accurately handle reconstruction biases, we show that the early emergence of *N. equitans *observed in previous analyses probably resulted from an LBA artifact due to the fast evolutionary rate of this archaeon, possibly worsened by LGT affecting a fraction of its ribosomal proteins. Indeed, the phylogenies based on our new ribosomal protein dataset and on additional single genes suggest that *N. equitans *is more likely to be a very divergent euryarchaeon - possibly a sister lineage of Thermococcales - than a new and ancestral archaeal phylum. This is consistent with further evidence gathered from close BLAST hits analyses on the whole genome complement of this taxon.

## Results and discussion

### Phylogenetic analysis of concatenated ribosomal proteins

Fifty ribosomal proteins having a sufficient taxonomic sampling and for which no LGT were evidenced in previous analyses (see Materials and methods and Table 1) [9,10] were concatenated into a large dataset (F1 dataset) comprising 6,384 positions and 25 archaeal taxa. The datasets contained 18 taxa previously used for the study of archaeal phylogeny based on ribosomal proteins [10] plus seven new taxa: the Thermococcale *Thermococcus gammatolerans*, the Methanomicrobiale *Methanogenium frigidum*, the Methanosarcinales *Methanococcoides burtonii*, *Methanosarcina mazei *and *Methanosarcina acetivorans*, the halobacterium *Haloferax volcanii *and *N. equitans*. Exhaustive maximum likelihood searches were performed with a Jones Taylor Thornton (JTT) model and limited constraints on indisputable nodes as recovered in unconstrained maximum likelihood and neighbor-joining analyses (data not shown) and in previous work [10].

**Figure 1:**
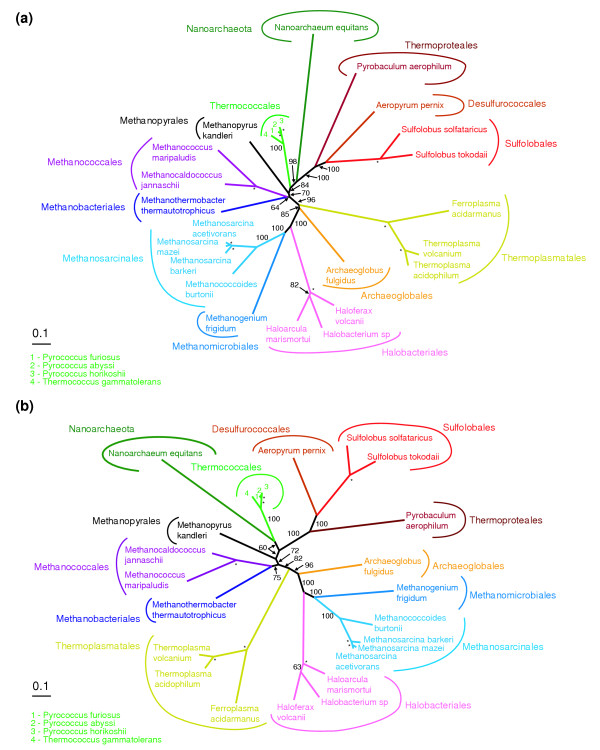
Unrooted maximum likelihood trees from exhaustive searches based on the F1 and the F2 datasets. **(a) **F1 dataset; **(b) **F2 dataset. Numbers at nodes are bootstrap values. Scale bars represent the number of changes per position for a unit branch length. Asterisks indicate constrained nodes.

The corresponding maximum likelihood unrooted tree is shown in Figure 1a. The monophyly of the two main archaeal domains, Crenarchaeota and Euryarchaeota, was recovered and supported by high bootstrap values (BV) (100% and 98%, respectively). Within the Euryarchaeota, the basal branching of Thermococcales (including *T. gammatolerans*) was also recovered (BV = 84%) as was the group comprising Methanobacteriales and Methanococcales (BV = 64%), and a well sustained group (BV = 96%) comprising Thermoplasmatales, Archaeoglobales, Halobacteriales (including *H. volcanii*) and Methanomicrobia (including the three new members of the Methanosarcinales *M. acetivorans*, *M. mazei*, *M. burtonii *and the Methanomicrobiale *M. frigidum*). *N. equitans *emerged as a separate branch distinct from those leading to Crenarchaeota and Euryarchaeota, in agreement with the rooted phylogeny of Waters and colleagues [13]. However, in our analysis the branch leading to *N. equitans *was relatively long, suggesting a possible above-average substitution rate with respect to the other taxa in the dataset that may affect its correct placement. Consequently, in order to identify the origin of possible biases in our global analysis, we analyzed two additional fusion datasets, one including the 27 proteins of the F1 dataset belonging to the large ribosomal subunit (F2 dataset) and one including the 23 proteins of the F1 dataset belonging to the small ribosomal subunit (F3 dataset).

The F2 tree (Additional data file 1A) was highly consistent with the F1 tree (Figure 1a) including the placement of *N. equitans *on a separate branch with respect to the other two archaeal domains. In contrast, in the F3 tree (Additional data file 1B), *N. equitans *emerged within the Euryarchaeota with a high statistical confidence (BV = 98%) and was supported - albeit weakly - as sister group of the Thermococcales (BV = 54%). This indicates that the components of the two ribosomal subunits may harbor a conflicting signal for the placement of *N. equitans*. Such incongruence was unexpected and led us to question the reliability of global ribosomal protein fusions in the assignment of the correct phylogenetic position of *N. equitans *in the archaeal phylogeny.

### Phylogenetic analyses of individual ribosomal proteins

To further characterize the conflicting phylogenetic signal for the placement of *N. equitans *in our concatenated analyses, we investigated its position in individual trees obtained by both unconstrained maximum likelihood and Bayesian analysis of each of the 50 ribosomal proteins. The topologies of these trees were consistent overall, despite the weakness of the phylogenetic signal contained in individual ribosomal proteins, often of small size. *N. equitans *generally displayed above-average branch lengths in these phylogenies, reinforcing the idea that LBA may strongly bias its placement in the global fusion trees. Moreover, *N. equitans *showed a highly unstable position (Table 1). In fact, it emerged as a separate branch distinct from the crenarchaeal and euryarchaeal domains (as in the F1 and F2 trees, Additional data file 1A), in only seven ribosomal protein phylogenies.

This is at odds with the indication of *N. equitans *as the representative of a novel archaeal domain, as Euryarchaeota and Crenarchaeota were generally well segregated in these individual phylogenies (data not shown). In contrast, as many as 33 ribosomal proteins supported the inclusion of *N. equitans *within the Euryarchaeota, 13 of which indicated a sister grouping with Thermococcales, similarly to the small ribosomal subunit protein tree (F3, Additional data file 1B). This striking affiliation may be explained by the occurrence of massive LGT involving these proteins between *N. equitans *and other euryarchaeal lineages. However, as no specific ecological reasons may especially favor such exchanges, this would rather indicate *N. equitans *as a euryarchaeal phylum rather than a novel archaeal domain. Conversely, LGT could easily explain the grouping of *N. equitans *with Crenarchaeota in the individual trees of nine ribosomal proteins (Table 1), as the genes coding for these proteins in *N. equitans *may have been acquired from its crenarchaeal host *Ignicoccus *species. If confirmed by future analyses, especially once the complete genome sequence of the *Ignicoccus *species is available, this would be the first report of numerous LGTs involving ribosomal proteins between two archaeal species.

It is worth noting that five of the nine proteins grouping *N. equitans *with Crenarchaeota belong to the large ribosomal subunit, and may introduce a strong bias for the basal position of *N. equitans *in the F2 tree (Additional data file 1A), as well as in the F1 tree (Figure 1a). To test this, we constructed a fourth dataset (F4 dataset) by removing these nine ribosomal proteins from the F1 dataset, and the resulting maximum likelihood tree is shown in Figure 1b. Strikingly, the F4 tree was highly consistent with the F1 tree, except for the position of *N. equitans*, which was strongly assigned to Euryarchaeota (BV = 100%) and branched off as a sister lineage of Thermococcales (BV = 60%), similarly to the small ribosomal subunit protein tree (F3, Additional data file 1B). Importantly, this placement is not likely to be the result of an LBA between the branch leading to *N. equitans *and that leading to Thermococcales, since the latter was rather short (Figure 1b). Our results strongly suggest that the basal position of *N. equitans *observed in our global ribosomal protein fusion analysis (Figure 1a) and in others [13] could resulted from the combination of conflicting phylogenetic signal from different subsets of ribosomal proteins (Table 1), either due to LGT and/or to LBA given the relatively fast evolutionary rates displayed by this taxon. Instead, once these biases are reduced, *N. equitans *shows a weak but specific affinity to Thermococcales (Figure 1b) that may represent its genuine placement in the archaeal phylogeny.

### Phylogenetic pattern of *N. equitans *protein complement

We investigated whether the difficulty of assigning the ribosomal proteins of *N. equitans *to a clear phylogenetic status reflected a general characteristic of the whole protein complement of this taxon. With this aim, we performed a complete survey of all 563 open reading frames (ORFs) encoded in the *N. equitans *genome by BLASTP searches against all other available complete archaeal genomes (including *T. gammatolerans*). Although a close hit does not always correspond to the nearest phylogenetic neighbor [22], a genome-scale analysis of the distribution of such hits can highlight interesting patterns. We have chosen not to extend this analysis further by automated molecular phylogeny reconstructions because we reckon that such an approach is highly prone to error. Indeed, dataset assembly is strictly dependent on human judgment at critical steps such as choice of homologs and alignment editing.

**Figure 2:**
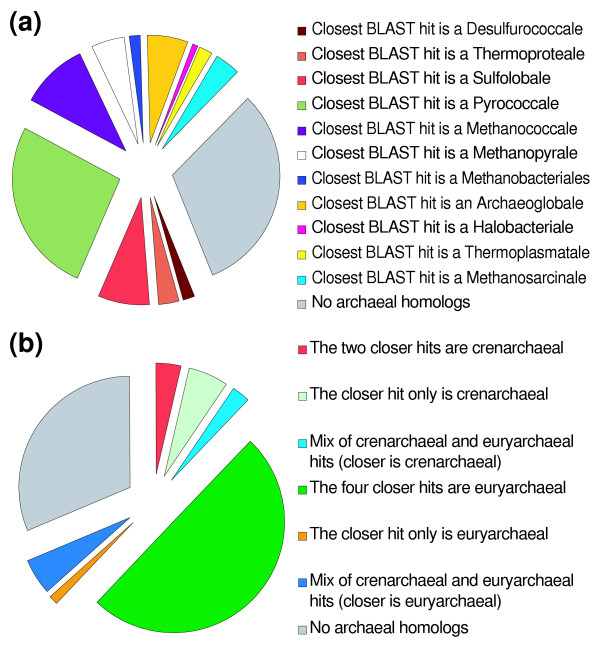
Distribution of close BLASTP hits. Hits are displayed as **(a) **per lineage and **(b) **per archaeal domain of the 563 ORFs of the *N. equitans *genome with a threshold of 10^-4^.

The distribution of close hits for the *N. equitans *ORFs according to an E-value cutoff of 10^-4 ^is shown in Figure 2a. Thresholds between 10^-2 ^and 10^-10 ^either increased or decreased the proportion of *N. equitans*-specific genes, but did not significantly change the relative distribution of close BLAST hits between archaeal groups (data not shown). A third of the *N. equitans *ORFs appeared to have no homologs in other archaea (gray section in Figure 2a), consistent with a previous analysis [13]. However, the remaining ORFs displayed many more close hits with different euryarchaeal lineages (56%) than with crenarchaeal ones (12%) (Figure 2a). Strikingly, nearly half of the euryarchaeal close hits (approximately 25% of the *N. equitans *ORFs) were represented by Thermococcales (green section in Figure 2a).

To identify possible biases introduced by LGT, we determined the global distribution of the second, third and fourth close BLAST hits (Figure 2b). Fifty percent of *N. equitans *close hits were indeed represented exclusively by members of different euryarchaeal phyla (green section in Figure 2b), and this proportion was even higher when we included ORFs with a crenarchaeon as close hit, but euryarchaeal species as next three close hits, suggesting possible Euryarchaeota-to-Crenarchaeota LGT (pale-green section in Figure 2b). Such a high fraction of close hits with the Euryarchaeota may be due to the effect of overall higher evolutionary rates in Crenarchaeota, although this has never been proposed. This unexpected high proportion of best close hits with Euryarchaeota - and notably Thermococcales - for the proteins of *N. equitans *is strikingly consistent with the phylogenetic analyses of individual (Table 1) and concatenated (Figure 1b and Additional data file 1B) ribosomal proteins, further suggesting that *N. equitans *may be a divergent euryarchaeon related to Thermococcales.

### Additional single-gene phylogenies

**Figure 3:**
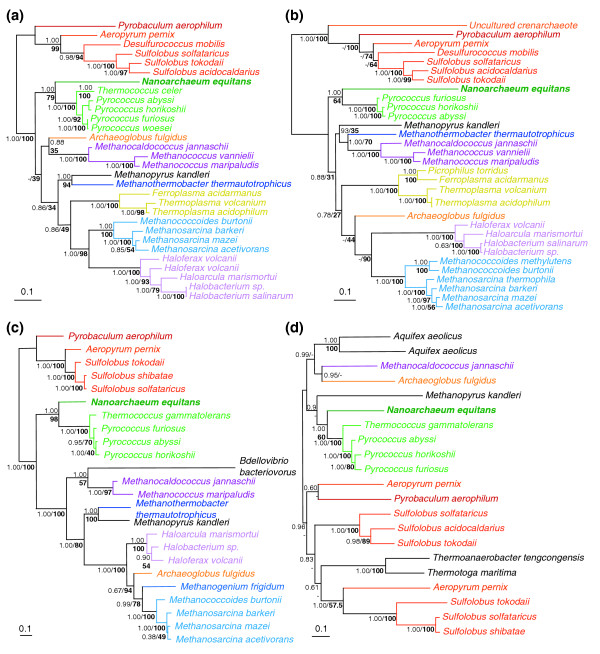
Phylogenetic trees for elongation factors EF-1α and EF-2, subunit A of topoisomerase VI and reverse gyrase. Unconstrained unrooted maximum likelihood trees of **(a) **elongation factor EF-1α, **(b) **elongation factor EF-2, **(c) **subunit A of topoisomerase VI, and **(d) **Bayesian tree of reverse gyrase. Bold numbers at nodes are bootstrap values; the other numbers are the Bayesian posterior probabilities. Scale bars represent the number of changes per position for a unit branch length.

To test further the phylogenetic position of *N. equitans*, we performed single-gene analyses by both maximum likelihood and Bayesian approaches of additional proteins known to be potential good molecular markers. Two unrooted archaeal maximum likelihood trees based on the elongation factors EF-1α and EF-2 are shown in Figure 3a and 3b, respectively. Strikingly, both trees strongly placed *N. equitans *within the Euryarchaeota (BV = 100% and a posterior probability (PP) of 1.00), and specifically as a sister-group of Thermococcales (BV = 79%, and PP = 1.00 and BV = 64% and PP = 1.00 in EF-1α and EF-2 trees, respectively), consistently with the F3 and F4 trees (Additional data file 1B and Figure 1b, respectively). The inclusion of *N. equitans *within the Euryarchaeota in the phylogeny based on EF-1α is further supported by an insertion/deletion (indel)-containing region that displays identical structure in *N. equitans *and several euryarchaeal lineages including Thermococcales (data not shown). These results may be interpreted by positing the concerted LGT of EF-1α and EF-2 from Thermococcales to *N. equitans*, since the two factors are part of the same macromolecular complex.

Thus, we analyzed additional markers involved in different molecular functions, such as the A subunit of topoisomerase VI, a type IIB DNA topoisomerase involved in DNA replication and whose phylogeny is highly consistent with that based on 16S rRNA [23]. The resulting tree (Figure 3c) was largely congruent with the previous ones, and once more placed *N. equitans *as sister-group of Thermococcales (BV = 98%, PP = 1.00), within the Euryarchaeota (BP = 100%, PP = 1.00). Finally, we investigated the position of *N. equitans *in an archaeal phylogeny based on reverse gyrase, a key enzyme composed of two domains, a helicase and a topoisomerase [24] and specific to thermophiles, where it catalyzes DNA positive supercoiling [25]. In *N. equitans *the gene encoding reverse gyrase is split into two noncontiguous coding sequences encoding the helicase and topoisomerase functions, respectively [13]. This has been taken as evidence for an ancestral nature of the reverse gyrase gene of *N. equitans*, consistent with the supposedly early emergence of this taxon [13]. However, the phylogeny of reverse gyrase (Figure 3d) supports a late branching of *N. equitans*, and surprisingly once more grouped with Thermococcales (BV = 60% and PP = 1.00). This suggests that the fission of the reverse gyrase gene in *N. equitans *probably resulted from a secondary event. Indeed, a high number of split genes appear to be a general feature of the *N. equitans *genome [13], as well as of those of fast-evolving archaeal taxa, such as *Methanopyrus kandleri *[26].

## Conclusion

The description of *N. equitans *by Huber and colleagues little more than two years ago marked an important step in our knowledge of the diversity and evolution of the Archaea, still the most unexplored of life's three domains. Indeed, *N. equitans *represents an example of symbiotic/parasitic life style between two archaeal species that is unprecedented [11,12]. The exceptionality of this archaeon was confirmed by the sequencing of its genome, which combines a minimal size close to the theoretical limits of a living cell with a stability not observed in other highly reduced genomes [13].

Despite all these characters indicating *N. equitans *as the member of a highly divergent lineage, we feel that its assignment to a novel archaeal phylum - the Nanoarchaeota - other than the well established Euryarchaeota and Crenarchaeota may be premature. Indeed, the distinctiveness of the *N. equitans *SSU rRNA primary structure may be an idiosyncrasy of this taxon due to a unique combination of adaptation to hyperthermophily and genome reduction. Our phylogenetic analyses of ribosomal proteins consistently show that *N. equitans *does not behave like the Euryarchaeota or the Crenarchaeota, which generally form clearly distinct branches in the archaeal tree, but shows instead a highly unstable placement. Similarly, the suggestion that *N. equitans *may represent an ancient divergence in the archaeal domain is far from being settled. In fact, the branching point of *N. equitans *is largely unresolved in the SSU rRNA phylogeny [12], and its basal placement in a recent tree of a ribosomal protein concatenation may be biased by the attraction of the long branches leading to *N. equitans *and to the eukaryotic sequences used as the outgroup [13]. Indeed, our unrooted phylogenies underline the above-average evolutionary rate of *N. equitans *and warn against the unreliability of global ribosomal protein fusions in assessing the correct placement of this taxon, because of LBA. Moreover, an additional bias may be introduced by LGT, as we suggest that a substantial fraction of *N. equitans *ribosomal proteins may have been exchanged with its crenarchaeal host. Our results indeed indicate an unsuspected close affinity of *N. equitans *with the Euryarchaeota, and notably with Thermococcales. This evidence is strongly reinforced by the specific and strong affinity of *N. equitans *with Thermococcales in trees of diverse molecular markers that do not lie in close proximity in the *N. equitans *genome, and on close BLAST hit analyses on the whole genome complement of this taxon. To explain all these findings, the most parsimonious explanation would be that *N. equitans *is a highly divergent euryarchaeal lineage possibly related to Thermococcales.

The hypothesis of nanoarchaea being a euryarchaeal lineage has important implications for our understanding of archaeal evolution, as characters in common between *N. equitans *and Euryarchaeota could be more easily considered as synapomorphies of the group rather than ancestral traits that would have been lost in the branch leading to Crenarchaeota. The characterization and genomic analysis of additional nanoarchaeal species will be necessary to confirm a specific affinity to Thermococcales, and to shed further light on the evolution of this intriguing group of archaea.

## Materials and methods

### Sequence retrieval and dataset construction

We updated a dataset of 62 ribosomal proteins from previous work [9,10]. In addition to *N. equitans *[11], we included six new taxa: two Methanosarcinales (*Methanosarcina mazei *[27] and *Methanosarcina acetivorans *[28]) whose complete genomes have been recently made available in public databases [29,30], and four other archaeal species whose genome sequencing is under way, that is, the Methanomicrobiale *Methanogenium frigidum *[31], the Methanosarcinale *Methanococcoides burtonii *[32], the Halobacteriale *Haloferax volcanii *[33], and the Thermococcale *Thermococcus gammatolerans *[34] (Y.Z. and F.C., unpublished work). Sequences were retrieved using BLASTP [35] at NCBI for *N. equitans*, *M. acetivorans *and *M. mazei*, and by TBLASTN [35] at the genome-sequencing website for *H. volcanii *[36], and at the draft genome analysis website [37] for *M. burtonii *[38] and for *M. frigidum *[38]. Unlike Waters and colleagues [13], and like our previous studies [9,10], we did not include any eukaryotic outgroup, in order to prevent LBA. Novel sequences were manually added to previous alignments [39] and ambiguous regions were removed.

Single alignment datasets were constructed for each of the 62 ribosomal proteins. From these, four concatenated datasets were constructed: one including 50 ribosomal proteins for which no LGT was evidenced in previous analyses and had a sufficient taxonomic sampling (at least 21 taxa) (F1 dataset); one including the 27 proteins from the F1 dataset belonging to the large ribosomal subunit (F2 dataset); one including the 23 proteins from the F1 dataset belonging to the small ribosomal subunit (F3 dataset); and one corresponding to the F1 dataset excluding nine ribosomal proteins supporting a close relationship between *N. equitans *and the Crenarchaeota (see Results and discussion) (F4 dataset). Four additional single alignment datasets were similarly constructed for the two elongation factors EF-1α and EF-2, the A subunit of topoisomerase VI (TopoVIa), and reverse gyrase.

### Phylogenetic analyses

To handle rate variation among sites, maximum likelihood-distance matrices (JTT model with a Gamma-law and eight discrete classes) were computed with TREE-PUZZLE [40] and used for neighbor-joining tree reconstruction by the NEIGHBOR program of the PHYLIP package [41]. Unconstrained maximum likelihood trees were computed using PHYML and the same parameters [42]. Bayesian phylogenetic trees were constructed using MrBayes [43] with a mixed model of amino-acid substitution and a Gamma-law (eight discrete classes). MrBayes was run with four chains for 1 million generations and trees were sampled every 100 generations. Exhaustive maximum likelihood searches were performed using the PROTML program of the MOLPHY package [44] with a JTT model and limited constraints on indisputable nodes as recovered in unconstrained maximum likelihood and neighbor-joining analyses and previous work [10]. Branch lengths and likelihoods for the 2,000 top-ranking topologies were computed using a JTT model including a Gamma-law and eight discrete classes with TREE-PUZZLE [40]. Bootstrap analyses were performed on 1,000 replicates using PUZZLEBOOT [45] and extended majority rule consensus trees were inferred with CONSENSE from the PHYLIP package [46]. All datasets and corresponding phylogenetic trees are available on request from C.B.

### Close BLAST hit analyses

All the ORFs of the *N. equitans *genome were retrieved from NCBI. For each ORF a BLASTP search was performed locally on a database of complete archaeal genomes including *T. gammatolerans*. Different distributions of close BLAST hits were manually established with E-value threshold cutoffs ranging from 10^-2 ^to 10^-10^. The same criteria were used to establish additional distributions including information from the next three close-hit representatives of different phyla. For example, when the first six close hits were represented by *T. gammatolerans*, *Pyrococcus abyssi*, *P. horikoshii*, *P. furiosus*, *M. kandleri *and *Sulfolobus solfataricus*, we considered as three first close BLAST hits Thermococcales, Methanopyrales and Sulfolobales.

## Additional data files

Additional data are available with the online version of this article. Additional data file 1 contains a figure showing unrooted unconstrained maximum likelihood trees computed by PHYML from a concatenation of large subunit and small subunit ribosomal proteins.

## Supplementary Material

Additional File 1 Numbers at nodes are bootstrap values. Scale bars represent the number of changes per position for a unit branch length Click here for file

## Figures and Tables

**Table 1:** Position of *Nanoarchaeum equitans *in maximum likelihood and Bayesian phylogenies of individual ribosomal proteins

Position of *N. equitans*	Proteins	Total
Basal position	L3, L10, L11, L31e, S5, S19e, S24e	7
		
Within Crenarchaeota and sister group to:		9
Sulfolobales	L16, L18e, L23	3
*Aeropyrum pernix*	S17e	1
*Pyrobaculum aerophilum*	L6, L20a, L29, S6e	4
Other	S10	1
		
Within Euryarchaeota and sister group of:		33
Thermococcales	L1, L2, L14, L15, L21e, L24, L32e, L37e, S3, S7, S17, S19, S28e	13
		
*Methanopyrus kandleri*	L4, L13, S13	3
Methanococcales	L18	1
*Methanothermobacter thermautotrophicus*	S4, S11	2
*Archaeoglobus fulgidus*	S8e, S9	2
Thermoplasmatales	L22, L30, S2, S3ae, S15	5
Methanomicrobiales	S8	1
Halobacteriales	S27a	1
Other	L5, L19e, L24e, S4e, S27e	6
		
Absent in *N. equitans*	L39e	1
