# Supplementary material for: Nanoarchaea: representatives of a novel archaeal phylum or a fast-evolving euryarchaeal lineage related to Thermococcales?
Source: Genome Biol. 2005 Apr 14;6(5):R42. doi: 10.1186/gb-2005-6-5-r42 (PMC1175954; doi:10.1186/gb-2005-6-5-r42)
Supplement: Additional File 1 — Numbers at nodes are bootstrap values. Scale bars represent the number of changes per position for a unit branch length [file gb-2005-6-5-r42-S1.pdf]

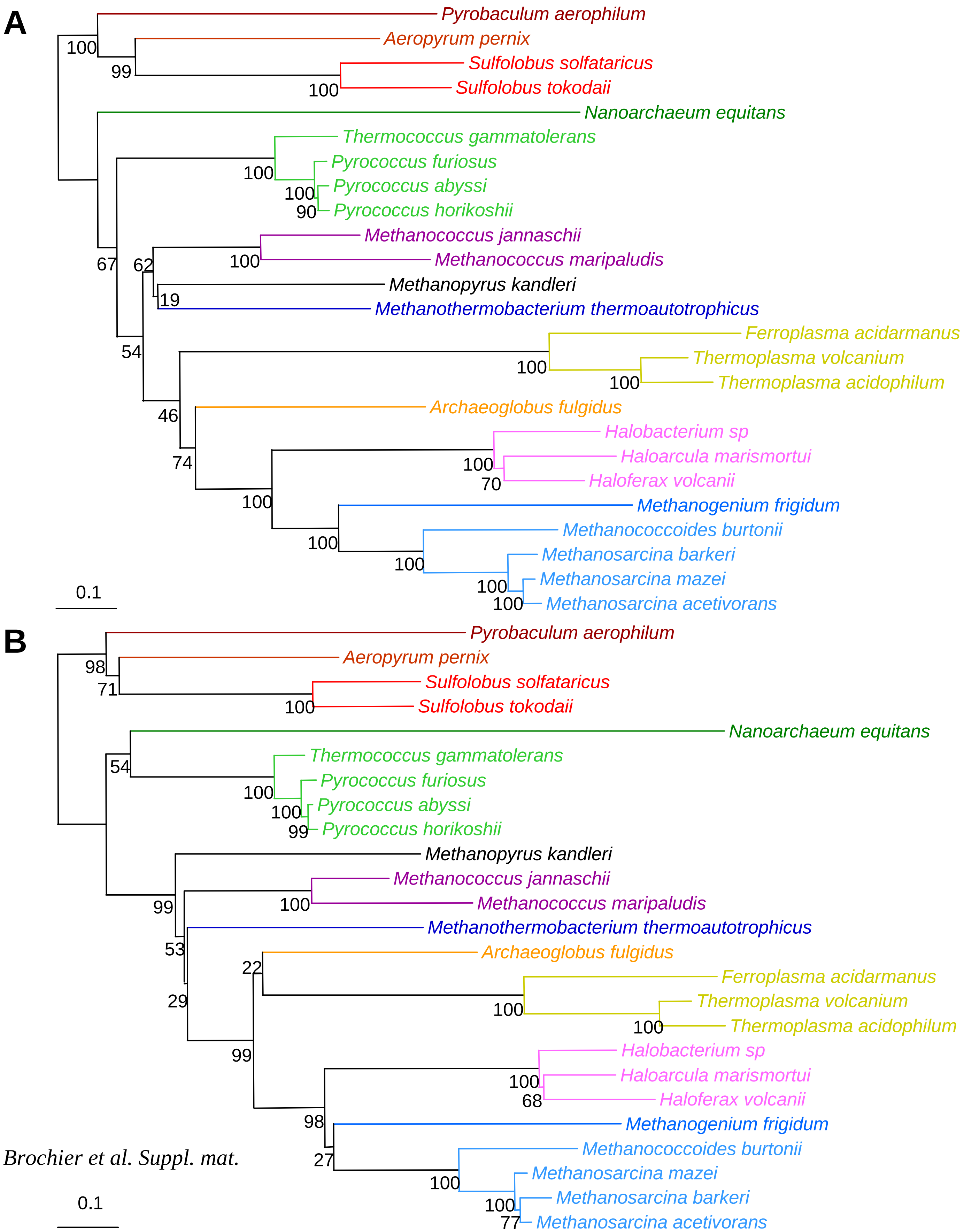

Brochier et al. Suppl. mat.

Unrooted unconstrained ML trees computed by PHYML from a concatenation of large subunit (A) and small subunit (B) ribosomal proteins. Numbers at nodes are bootstrap values. Scale bars represent the number of changes per position for a unit branch length.
